# Computational identification of genetic subnetwork modules associated with maize defense response to Fusarium verticillioides

**DOI:** 10.1186/1471-2105-16-S13-S12

**Published:** 2015-09-25

**Authors:** Mansuck Kim, Huan Zhang, Charles Woloshuk, Won-Bo Shim, Byung-Jun Yoon

**Affiliations:** 1 Department of Electrical and Computer Engineering, Texas A&M University, College Station, TX, USA; 2 Department of Plant Pathology & Microbiology, Texas A&M University, College Station, TX, USA; 3 Department of Botany & Plant Pathology, Purdue University, West Lafayette, IN, USA; 4 College of Science and Engineering, Hamad bin Khalifa University (HBKU), Doha, Qatar

**Keywords:** Maize, *Fusarium verticillioides*, host-pathogen interaction, network-based analysis, subnetwork module identification

## Abstract

**Background:**

Maize, a crop of global significance, is vulnerable to a variety of biotic stresses resulting in economic losses. *Fusarium verticillioides *(teleomorph *Gibberella moniliformis*) is one of the key fungal pathogens of maize, causing ear rots and stalk rots. To better understand the genetic mechanisms involved in maize defense as well as *F. verticillioides *virulence, a systematic investigation of the host-pathogen interaction is needed. The aim of this study was to computationally identify potential maize subnetwork modules associated with its defense response against *F. verticillioides*.

**Results:**

We obtained time-course RNA-seq data from B73 maize inoculated with wild type *F. verticillioides *and a loss-of-virulence mutant, and subsequently established a computational pipeline for network-based comparative analysis.

Specifically, we first analyzed the RNA-seq data by a cointegration-correlation-expression approach, where maize genes were jointly analyzed with known *F. verticillioides *virulence genes to find candidate maize genes likely associated with the defense mechanism. We predicted maize co-expression networks around the selected maize candidate genes based on partial correlation, and subsequently searched for subnetwork modules that were differentially activated when inoculated with two different fungal strains. Based on our analysis pipeline, we identified four potential maize defense subnetwork modules. Two were directly associated with maize defense response and were associated with significant GO terms such as GO:0009817 (defense response to fungus) and GO:0009620 (response to fungus). The other two predicted modules were indirectly involved in the defense response, where the most significant GO terms associated with these modules were GO:0046914 (transition metal ion binding) and GO:0046686 (response to cadmium ion).

**Conclusion:**

Through our RNA-seq data analysis, we have shown that a network-based approach can enhance our understanding of the complicated host-pathogen interactions between maize and *F. verticillioides *by interpreting the transcriptome data in a system-oriented manner. We expect that the proposed analytic pipeline can also be adapted for investigating potential functional modules associated with host defense response in diverse plant-pathogen interactions.

## Introduction

Maize is one of the most significant crops in the world. Unfortunately maize is susceptible to a variety of pathogens, resulting in economic losses. *Fusarium verticillioides *is an important fungal pathogen causing maize ear and stalk diseases. More alarmingly, the fungus produces fumonisins, a group of toxic secondary metabolites harmful to animals and humans [1,2]. Unfortunately, the development of effective management strategies for diseases has been stagnant due to a lack of basic understanding of this complicated host-pathogen interaction. The majority of, if not all, plant-microbe interactions that result in severe economic damages are cryptic and difficult to comprehend even with today's technological advancements. Plants respond to a variety of external stimuli, in particular microbial pathogens, with sophisticated response mechanisms, which we have recently started to gain a deeper understanding of. In contrast to the adaptive immune system found in animal systems, plant defense systems encode groups of genes to recognize and respond to specific pathogens [3,4]. On the other hand, plant-associated microbes have coevolved with their hosts to overcome plant innate immunity and use a repertoire of effectors, enzymes and toxins to suppress host defense. Therefore, characterizing maize defense against *F. verticillioides *is critical for better comprehension of their mutual interactions as well as further enhancement of maize resistance.

Recently, the widespread application of high throughput technologies, such as microarray technology and next generation sequencing (NGS), has made a significant contribution to the study of host-pathogen interactions. In maize-pathogen interactions, several methods utilizing microarray data have focused on evaluating gene expression. For instance, Kelley *et al*. [5] identified maize genes involved in host resistance (or susceptibility) to pathogenic fungus *Aspergillus flavus *based on expression changes. Similarly, Campos-Bermudez *et al*. [6] identified maize genes and metabolites that showed expression variation after inoculation with *F. verticillioides*. Unfortunately, these analyses, as well as numerous other published studies, focused on expression differences in individual genes. For host-pathogen studies other than plant-pathogen, searching for host-pathogen gene pairs was performed through correlation analysis. Using Pearson's or Spearman rank correlation, Shea *et al*. [7] identified associated gene pairs in a human-bacterial system with Group A *Streptococcus *(GAS) and Reid *et al*. [8] identified molecular interactions between mouse and *Plasmodium *as well as mosquito and *Plasmodium*. In addition, Asters *et al*. [9] successfully constructed networks using Euclidean distance calculation based on their correlated gene pairs. While these approaches were successful in identifying correlated gene pairs and their networks in host-pathogen systems, improvements can be made with systematic investigation of cellular interactions or processes for underlying host-pathogen interactions. Network-based approaches [10-13] jointly analyzing gene expression data and protein-protein interaction (PPI) are receiving attention as better strategies for predicting biological markers or subnetwork modules. For example, Chuang *et al*. [10] predicted potential subnetwork modules associated with breast cancer metastasis using gene expression data as well as PPI network. Kim *et al*. [13] identified fungal virulence-associated subnetwork modules by seeking functionally coherent genes by using network-based comparative analysis.

In this study, our objective was to identify potential maize defense modules against the *F. verticillioides *in maize co-expression networks. We analyzed the RNA-seq data with the pipeline protocols and focused on the maize networks associated with maize-*F. verticillioides *interactions. Particularly, in addition to a wild type *F. verticillioides*, we used a *F. verticillioides *mutant, designated *fsr1 *strain, which shows a drastic reduction in fungal virulence [14]. Our hypothesis is that the mutation in *fsr1 *gene disrupts downstream genetic networks that are crucial for maize-*F. verticillioides *interaction, and by analyzing differentially regulated subnetwork modules we will be able to identify important disease resistance mechanisms in maize. We also considered the dynamic changes in gene expression during maize-*F. verticillioides *interaction, and designed our study to collect RNA-seq data from three colonization phases: establishment of fungal infection (3 days post inoculation [dpi]), colonization and movement in vascular bundle (6 dpi) and host destruction and collapse (9 dpi). Prior to our computational analyses, we selected representative *F. verticillioides *virulence genes and searched for corresponding candidate maize genes that might be potentially associated with maize defense response. We used a cointegration-correlation expression approach to compare time-course expression patterns between maize and the selected *F. verticillioides *genes to obtain candidate maize genes. Based on the maize candidates, we predicted maize co-expression networks by partial correlation and searched subnetwork modules not only differentially expressed in the two conditions (*i.e*., wild type infected vs. the mutant infected conditions), but also composed of harmoniously coordinated genes. In this detection process, subnetwork modules were extended by the computationally efficient branch-out technique [13] with the probabilistic pathway activity inference [14]. Based on the analysis, we identified potential maize subnetwork modules associated with maize defense response that were specifically defense-associated, well coordinated, and differently activated in the two conditions.

## Materials and methods

### Overview of the proposed host-pathogen interaction analysis pipeline

**Figure 1:**
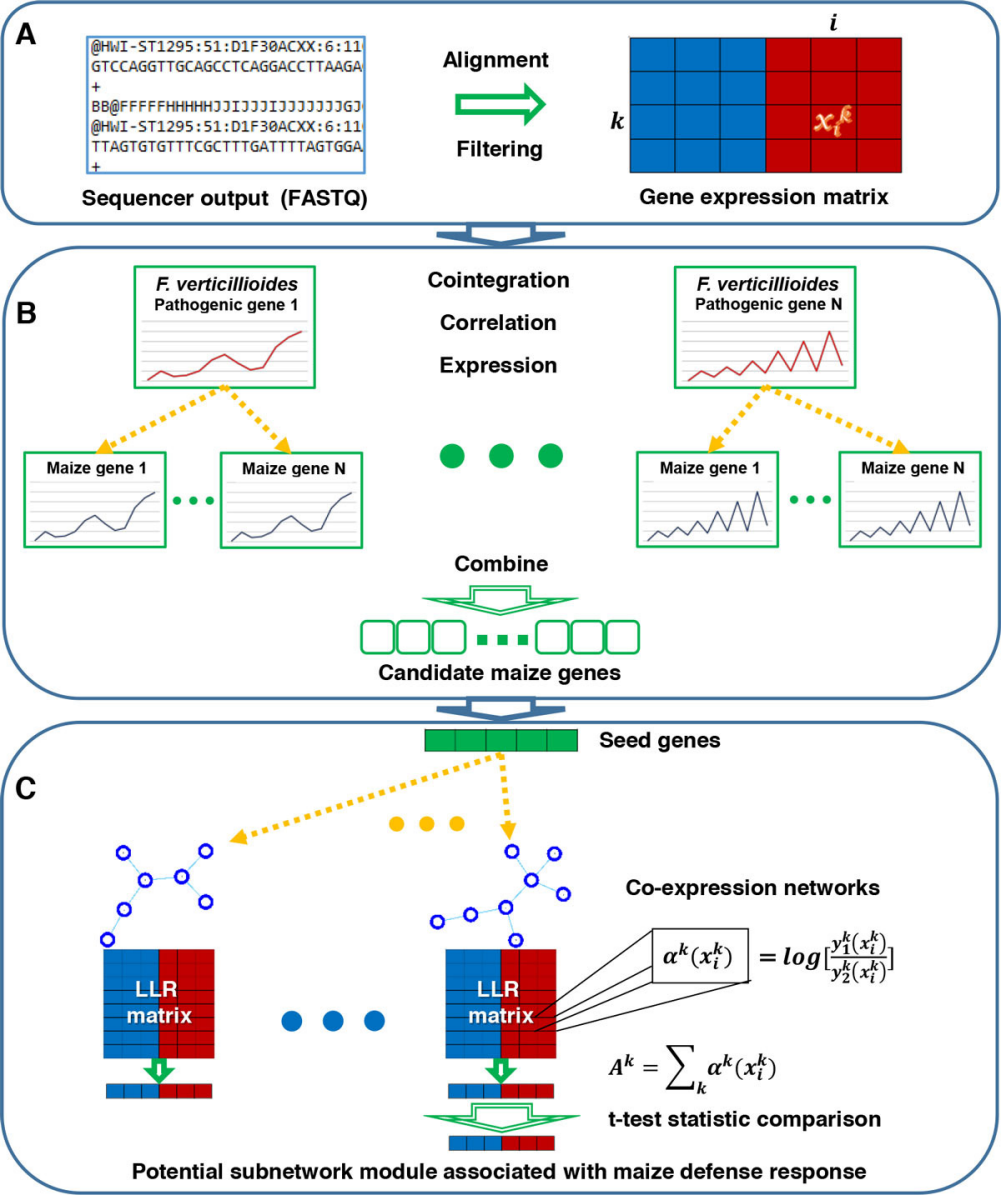
**Overview of the proposed network-based comparative analysis pipeline for predicting potential maize subnetwork modules associated with maize defense response**. 1st step: the RNA-seq data were preprocessed by aligning them to the reference genome and filtering out lowly expressed genes for quality control to obtain the gene expression matrix. 2nd step: in order to predict important candidate genes potentially involved in maize defense modules, a cointegration-correlation-expression approach was applied to identify maize genes, whose expression patterns correspond to those of selected *F. verticillioides *pathogenicity genes. 3rd step: co-expression networks surrounding the candidate maize genes were predicted and a log-likelihood ratio (LLR) matrix was computed for subsequent analysis. Through a seed-and-extend approach with an efficient branch-out technique, we searched for potential maize subnetwork modules starting from the top 20% differentially expressed seed genes. Finally, potential maize subnetwork modules involved in defense response were predicted by evaluating the strength of the association between the module activity level and the pathogenicity of the fungi.

Figure 1 provides an overview of the proposed pipeline for network-based comparative gene expression analysis, particularly focusing on maize-*F. verticillioides *interactions. As shown in Figure 1A, we first preprocessed the RNA-seq data by aligning them to the reference genomes and then filtered out genes with insignificant expression. The preprocessed gene expression data were analyzed in the next step shown in Figure 1B, where cointegration-correlation-expression analysis was performed to identify candidate maize genes whose expression patterns correspond to known *F. verticillioides *virulence genes. Figure 1C illustrates the third step of our pipeline, where the gene expression data of the candidate maize genes were used to construct co-expression networks of the maize genes based on partial correlation coefficients. Furthermore, the gene expression values were converted into log-likelihood ratios (LLRs) for subsequent analysis. Finally, based on the co-expression networks, maize subnetwork modules were identified by expanding the subnetwork regions around the top 20% differentially expressed genes, where we utilized a computationally efficient branch-out technique. As a result of our analysis, we identified four potential maize subnetwork modules possibly associated with maize defense response. The detailed description of each step in the analysis pipeline is provided in the following subsections.

### Sample preparation

Maize stalks were inoculated with *F. verticillioides *wild type and *fsr1 *mutant as previously described [14]. We used the inbred line B73, a major source of commercial maize hybrids, that has no known stalk rot resistance, for this study. Maize stalk samples were collected 3, 6, and 9 dpi using manual sectioning, and scanned with fluorescence microscopy to identify host tissue damage and/or fungal colonization, particularly in the vascular bundles. These samples were dissected and collected separately for RNA extraction and cDNA synthesis following standard molecular biology procedures. For each sample subjected to sequencing, sectioning was performed on at least three stalk samples from each stage of infection, and isolated tissues were pooled for RNA extraction.

### RNA sequencing and preprocessing

Figure 1A illustrates the first step of our analysis pipeline, where we preprocessed the RNA-seq data to obtain the normalized gene expression matrix. As previously described [13], RNA sequencing was processed using Illumina HiSeq 2000 producing sequencing results with relatively high quality and high coverage. In this sequencing, library preparation and RNA isolation were performed by Illumina's simplified sample prep kits and small RNA sample preparation kit, respectively. Also, Illumina HCS 1.515.1 and RTA 1.1348.0 also performed quality prefiltering, uncertainty assessment, base calling, and sequence cluster identification simultaneously. For *F. verticillioides *as well as maize, six independent sequencing libraries at three time points (*i.e*., 3 dpi, 6 dpi, and 9 dpi) for both wild type-inoculated and the mutant-inoculated samples, were obtained so that a total of 36 sample libraries were prepared. The prepared reads from the 36 libraries were mapped to the *F. verticillioides *strain 7600 reference genome [15] and to the maize B73 genome [16]. The alignment was performed by Bowtie2 [17] and another NGS data analysis tool called Sam-tools [18] was used to analyze the alignment result and obtain the read counts of all *F. verticillioides *genes as well as maize genes. In the alignment process, Bowtie2, optimized for gapped alignment and relatively longer reads, performed end-to-end mapping and extracted SAM format files by default mode. Next, filtering out genes with insignificant expression left 57% of *F. verticillioides *genes (8,072 genes) and 42.2% of maize genes (57,676 genes) for our subsequent analysis. During the filtering process, genes expressed in less than half of the total replicates, were eliminated. However, genes that were expressed only in one of the two conditions (i.e., wild type inoculated vs. mutant inoculated) were kept when they were expressed in more than 70% of the replicates in a given condition. This filtering process was performed to ensure that we retain potentially important differentially expressed genes while removing barely expressed genes. Finally, the filtered NGS data were normalized for relative quantification. For normalization, every read count across all replicates was normalized by the corresponding gene length. It is worth noting that, at this stage, the expression level was not normalized across different time points.

Instead, such differences were analyzed in one of the following steps of our analysis pipeline through cointegration, which investigates the time-course evolution of the gene expression levels to identify *F. verticillioides *and maize genes that may be associated with each other. Table 1 shows the general statistics of our RNA-seq datasets prepared for the subsequent analysis. The table not only demonstrates the differences between the two *F. verticillioides *strains, but also indirectly illustrates how the virulence of *F. verticillioides *impacts maize transcription profile over time.

**Table 1:** General statistics of the RNA-seq datasets analyzed in the current study

		3dpi	Wild type 6dpi	9dpi 3dpi	Mutant 6dpi	9dpi
	Type of run			single		
	Read length			100 (bp)		
*Fusarium*	Mean number of reads aligned	82504	200827	235702 24711	63462	138406.3
*verticillioides*	median depth of coverage	5.8	14.2	16.6 1.8	4.5	9.8
	Mean number of reads aligned	4394510	4183565	3377730 3798777	3589860	3577877
Maize	median depth of coverage	32.1	30.6	24.7 27.8	26.2	26.2

### Selection of pathogenicity genes of *F. verticillioides*

In order to narrow down maize genes to key candidates likely involved in maize defense mechanism, we selected representative *F. verticillioides *virulence genes whose expression patterns over time would be used as the criterion in the comparison. Among known *F. verticillioides *virulence genes, we selected the following four genes for our analysis after carefully considering their expression patterns, biochemical and physiological functions, as well as evolutionary conservation in other pathogenic fungi; i) *FSR1 *(FVEG 09767) - associated with fungal stalk rot virulence and sexual mating [14]; ii) *FST1 *(FVEG 08441) - associated with fungal growth and development particularly on maize ears [19]; iii) *FvVE1 *(FVEG 09521) - associated with aggressive pathogenesis and toxin production on maize seedlings [20]; iv) *ZFR1 *(FVEG 09648) - an important transcription factor controlling fungal growth and toxin biosynthesis on maize kernels [21]. Once we established our preliminary network-based comparative analysis pipeline procedure, we were able to subsequently incorporate additional *F. verticillioides *virulence genes to improve the robustness of our prediction model.

### Cointegration-correlation-expression analysis

**Figure 2:**
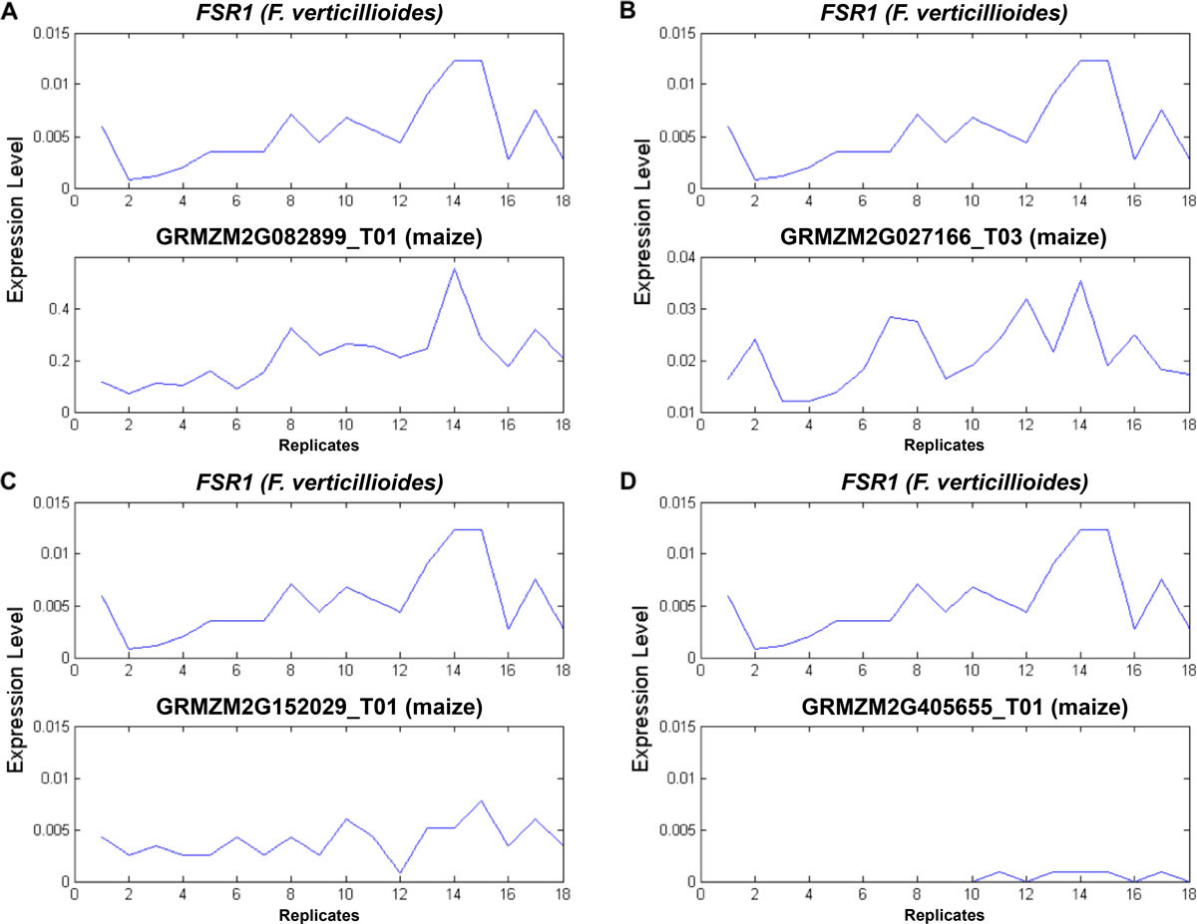
**Four practical examples in comparing the expression patterns between maize genes and FSR1, one of the selected pathogenicity genes of F. verticillioides**. Y-axis corresponds to the normalized expression level and X-axis corresponds to the replicate ID (replicates 1-6: 3 dpi, replicates 7-12: 6 dpi, replicates 13-18: 9 dpi). Four different cases are illustrated: A) *FSR1 *vs. GRMZM2G082899 T01 (cointegration: good, correlation: good, expression: good) - GRMZM2G082899 T01 was considered as a good corresponding maize gene to *FSR1 *; B) *FSR1 *vs. GRMZM2G027166 T03 (cointegration: good, correlation: bad, expression: good) - the association between the two genes turned out to be weak based on correlation; C) *FSR1 *vs. GRMZM2G152029 T01 (cointegration: bad, correlation: good, expression: good) - the time-course expression trends were dissimilar for the two genes, reflected in low cointegration; D) *FSR1 *vs. GRMZM2G405655 T01 (cointegration: good, correlation: good, expression: bad): GRMZM2G405655 T01 gene was not considered to be a good candidate, since it was hardly expressed.

Using the four representative *F. verticillioides *virulence genes, we performed a comprehensive analysis on tendency of expression levels for maize genes over time across all replicates to narrow down maize genes into prime candidates. This step is illustrated in Figure 1B, where we considered cointegration, correlation, and expression, so we can jointly analyze the expression levels of maize genes and selected *F. verticillioides *virulence genes. First, cointegration [22] was applied to track a long-run relationship of expression levels in the two species (*i.e*., maize vs. *F. verticillioides*), which are nonstationary and involve time-varying uncertainty. In this analysis, the expression levels of a maize gene and a virulence gene of *F. verticillioides *were cointegrated over time to see whether the given genes share any common expression trend across all replicates. The Engle-Granger method of cointegration identifying single cointegrating relations between the host and the pathogen was applied. For each representative virulence gene of *F. verticillioides*, maize genes whose *p*-value of the Engle-Granger test was less than 0.05 were taken into account as candidates. Second, correlation was used to trace expression patterns of the two species over all replicates. It quantified the strength of a linear relationship between maize genes and each representative virulence gene of *F. verticillioides *across all replicates over all time points. Corresponding maize genes whose Pearson's correlation coefficient was higher than 0.65 (*p*-values less than 0.0035) [Additional file 1: Table S1] to a *F. verticillioides *virulence gene were considered as candidates. Third, expression levels of maize genes over all replicates were monitored in order to remove the genes that were barely expressed in our dataset. Maize genes whose mean expression levels were in the top 80% of all genes, and non-zero in all replicates, were taken into consideration as candidates since the selected *F. verticillioides *virulence genes were also expressed in all replicates. Each selected *F. verticillioides *virulence gene was used as a criterion to search for candidate maize genes through this combined analysis of cointegration, correlation, and expression. Finally, candidate maize genes obtained by comparison with the four selected virulence genes were combined for subsequent analysis. In our analysis to identify candidate genes that may be involved in the maize defense mechanism, we set the *p*-value threshold for the Pearson's correlation and that for the Engle-Granger test such that, on average, 50% of the candidate genes identified based on a given pathogenicity gene were also among the candidates predicted by other pathogenicity genes. This reduces the dependence of our prediction results on a specific pathogenicity gene. Figure 2 illustrates the underlying motivation of the proposed combined approach based on several realistic examples. The figure demonstrates how the combined use of cointegration, correlation, and expression can lead to better prediction of candidate maize genes likely to be associated with the maize defense response. Specifically, each of the examples shown in Figure 2(B-D) illustrates the case when the gene under consideration does not meet one of the cointegration, correlation, expression criteria (while meeting the other two remaining criteria), therefore determined not to be a good candidate gene.

### Identification of maize subnetwork modules

In order to identify potential maize subnetwork modules associated with maize defense response, we adopted a similar approach that we have recently proposed in [13]. The method was used to predict pathogenic network modules in the co-expression network of *F. verticillioides*, constructed from RNA-seq data, and the method was shown to be capable of detecting potential modules that are biologically plausible. This step is illustrated in Figure 1C. Here, to identify genetic subnetwork modules possibly associated with maize defense response, we started by constructing co-expression networks of maize based on the candidate maize genes that were specifically selected against the *F. verticillioides *virulence genes through the cointegration-correlation-expression analysis. These co-expression networks were predicted based on partial correlation computed from the preprocessed gene expression matrix of the candidate maize genes. While constructing the co-expression networks, we excluded relatively weak interactions between the maize candidates. We constructed four different co-expression networks at four different threshold levels as shown in Table 2. As discussed in [13], the use of multiple co-expression networks predicted at different threshold levels can mitigate potential issues that may arise when relying on a specific threshold level. Given the four maize co-expression networks, we searched for "seed genes" in the networks that were significantly differentially expressed in the two conditions. More specifically, maize genes with top 20% *t*-test score for discriminating between the two conditions were selected as seed genes. Finally, we expanded the maize subnetwork modules in the co-expression networks starting from the selected seed genes, in order to identify potential genetic modules that are likely to be associated with the maize defense response. We first examined all the connected genes to each seed gene, and evaluated whether expanding the subnetwork module by adding one of the connected genes would enhance the discriminative power of the subnetwork module, if we were to probabilistically infer the module activity [23] and used it to differentiate between the two conditions. Subsequently, we continued to expand the subnetwork module by recruiting one of the neighboring genes into the module at a time, using a computationally efficient branch-out technique [13]. A neighboring gene was added if adding the gene improves the discriminative power of the subnetwork module (measured in terms of the *t*-test statistics score of the probabilistically inferred module activity) by at least 5%. Furthermore, at each extension step, the branch-out technique considered up to three subnetwork modules whose discriminative power were within 2% from the top. This process was repeated until there was no subnetwork module, whose discriminative power could be improved at least by 5% through extension. The overall process was repeated for all seed genes using all four co-expression networks. Finally, for the maize subnetwork modules identified by our network-based comparative analysis, we investigated whether the subnetwork modules were associated with significant GO terms. In this GO analysis, we selected those modules, at least 30% of whose member genes were annotated by significant GO terms. Significance of a GO term was assessed based on the *p*-value of the Benjamini-Hochberg false discovery rate (FDR) method [24] computed by g:Profiler (http://biit.cs.ut.ee/gprofiler/) [25]. The GO term was considered to be significant if the *p*-value was less than 0.05. Consequently, our final prediction of the potential maize defense subnetwork modules were made based on the strength of association between the module activity level and the two conditions under comparison (inoculated with wild type vs. *fsr1 *mutant) as well as their association with significant GO terms.

**Table 2:** Properties of the co-expression networks around the candidate maize genes (for maize inoculated with wild type *F.verticillioides *and maize inoculated with *fsr1 *mutant strain)

Threshold (cut-off partial correlation)	Wild type		Mutant	
	**number of genes**	**number of interactions**	**number of genes**	**number of interactions**
0.9	97	269	93	243
0.8	106	546	101	518
0.7	111	868	107	845
0.6	114	1257	111	1211

### Probabilistic subnetwork activity inference

As described in the previous subsection, to identify potential subnetwork modules, we used a seed-and-extend approach with a branch-out scheme, where the goodness of a given subnetwork module was evaluated by inferring the module activity and assessing its effectiveness in discriminating between the two different conditions. For this purpose, we adopted a probabilistic pathway activity inference scheme, which was originally proposed in [23] and was previously applied to the prediction of pathogenic gene modules in *F. verticillioides *[13]. In the following, we present a brief summary of the method. Suppose we have a set of genes *G *= {*g*_1_*, g*_2_*,..., g_n_*} that belong to a given subnetwork module and the expression levels of these genes are **x **= {*x*^1^*, x*^2^*,..., x^n^*}. The activity level of the given subnetwork module can be measured by

(1) $$\eta \left(\text{x}\right)=\overset{n}{\underset{k=1}{\sum }}\alpha^{k}\left(x^{k}\right),$$

where *α^k^*(*x^k^*) is the log likelihood ratio (LLR) between the two conditions (*i.e*., maize inoculated with two different strains - wild type vs. the mutant - of *F. verticillioides*) defined as follows

(2) $$\alpha^{k}\left(x^{k}\right)=\text{log}\left[\frac{y_{1}^{k}\left(x^{k}\right)}{y_{2}^{k}\left(x^{k}\right)}\right].$$

In equation (2), $y_{1}^{k}\left(x\right)$ is the conditional probability density function (PDF) of the expression level of gene *g_k _*in one condition. Similarly, $y_{2}^{k}\left(x\right)$ is the conditional PDF of the expression level of gene *g_k _*in the other condition. We can estimate the activity level of *η*(**x**) of the subnetwork module as defined in (1) and also assess its discriminative power for differentiating between the two different conditions using the *t*-test statistics score:

(3) $$t\left(g\right)=\frac{\mu_{1}-\mu_{2}}{\sqrt{\frac{s_{1}^{2}}{n_{1}}+\frac{s_{2}^{2}}{n_{2}}}},$$

where *µ*_1 _and $s_{1}^{2}$ are the mean and the variance of the subnetwork activity level in one condition, and *µ*_2 _and $s_{2}^{2}$ are the mean and the variance of the subnetwork activity level in the other condition. *n*_1 _and *n*_2 _are the number of replicates (or independent measurements) in the respective conditions. For further details, readers are referred to [23], where the method was originally proposed in the context of cancer classification.

## Results

### Characteristics of the candidate maize genes

Before predicting potential maize subnetwork modules involved in the maize defense response, we investigated whether our candidate maize genes obtained through the cointegration-correlation-expression analysis using the selected *F. verticillioides *virulence genes were possibly associated with maize defense. In order to examine whether the candidate maize genes were defense-associated, we compared them with the maize genes that corresponded to the *F. veticillioides *housekeeping genes, which are constitutively expressed and are mainly involved in the maintenance of fundamental cellular functions. For this comparison, four *F. verticillioides *housekeeping genes commonly used in molecular genetic studies were selected: the two beta-tubulin genes (FVEG 04081 and FVEG 05512), the pyruvate dehydrogenase E1 component subunit alpha gene (FVEG 07074), and the glyceraldehyde 3-phosphate dehydrogenase gene (FVEG 04927). For each *F. verticillioides *housekeeping gene, we searched for the corresponding maize genes through the cointegration-correlation-expression approach, and compared them against the candidate maize genes that were predicted to correspond to the four *F. verticillioides *virulence genes selected in our study. Both groups of *F. verticillioides *genes (housekeeping and virulence genes) were all relatively significantly expressed in all replicates, so one could presume that their expression patterns might be similar. However, the maize genes that were predicted to correspond to the *F. verticillioides *housekeeping genes and the candidate maize genes that corresponded to the *F. verticillioides *virulence genes overlapped only about 20% on average. Therefore, we concluded that the candidate maize genes that were identified by comparing maize genes against the selected *F. verticillioides *virulence genes using our proposed cointegration-correlation-expression approach were indeed very likely to be associated with the maize defense mechanism.

### Identification of potential maize defense subnetwork modules

**Figure 3:**
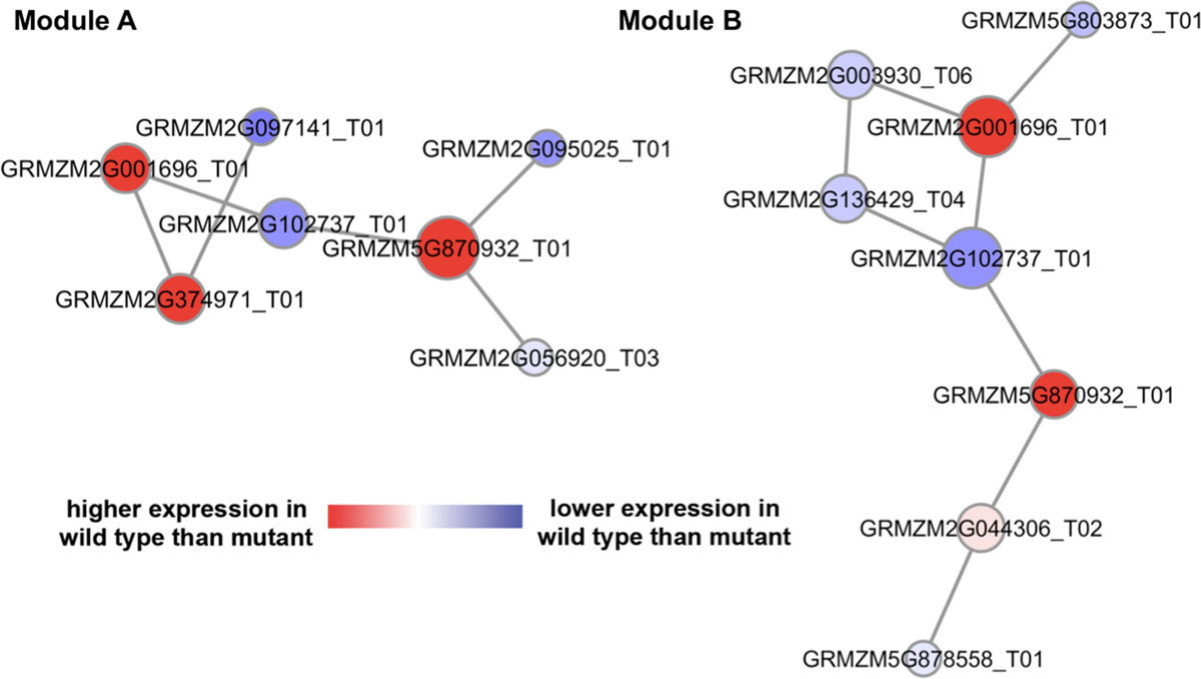
**Two potential maize subnetwork modules directly associated with maize defense response to fungi**. [Additional file 1: Table S2] i) Module-A comprised seven maize genes, where three of them - GRMZM2G001696 T01, GRMZM2G374971 T01, and GRMZM5G870932 T01 -were known maize genes annotated with a significant GO term GO:0009817 "defense response to fungus (incompatible interaction)" with a *p*-value of 1.25e-06. ii) Module-B was composed of eight maize genes, where three of them - GRMZM2G001696 T01, GRMZM5G870932 T01, and GRMZM5G878558 T01 - were known maize genes annotated with a significant GO term GO:0009620 "response to fungus" with a *p*-value of 5.69e-07.

**Figure 4:**
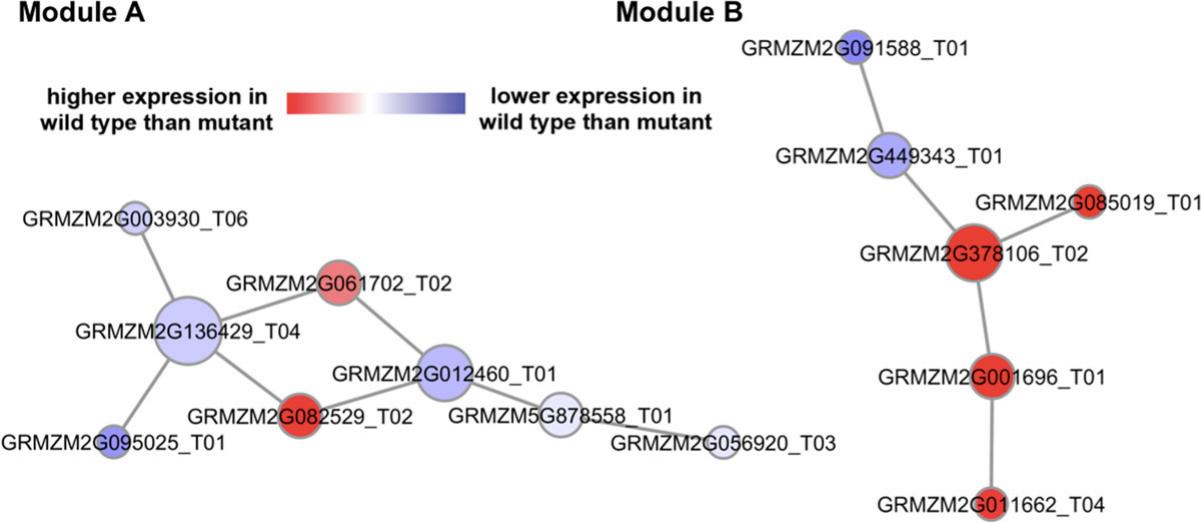
**Two additional potential maize subnetwork modules identified through the proposed network-based comparative analysis pipeline**. [Additional file 1: Table S3] i) Module-A comprised eight maize genes, where four of them - GRMZM2G003930 T06, GRMZM2G056920 T03, GRMZM2G095025 T01, and GRMZM5G878558 T01 - were known maize genes annotated with the GO term GO:0046914 "transition metal ion binding" with a *p*-value of 4.56e-02. ii) Module-B was composed of six maize genes, where two of them - GRMZM2G001696 T01 and GRMZM2G085019 T01 - were known maize genes annotated with the GO term GO:0046686 "response to cadmium ion" with a *p*-value of 3.00e-02.

Through the proposed network-based comparative RNA-seq data analysis pipeline, we identified four potential maize subnetwork modules associated with the maize defense response against *F. verticillioides*. The four identified potential maize defense subnetwork modules are illustrated in Figures 3 and 4. To search for potential maize defense subnetwork modules, we selected four representative *F. verticillioides *virulence genes and compared their expression patterns with those of maize genes in order to find significant maize gene candidates in such modules. Specifically, the cointegration-correlation-expression approach was applied to find the candidate maize genes whose expression trends are comparable with those of the selected *F. verticillioides *genes. Based on the candidate maize genes, we predicted co-expression networks around them, and further chose the top 20% significantly differentially expressed genes as seed genes. Starting from each of these seed genes, we iteratively extended the subnetwork module by recruiting additional neighboring genes whose inclusion enhanced the discriminative power of the module (measured by the *t*-test statistics score) by at least by 5%. Amongst the extended subnetworks (with one additional neighboring gene), we selected the subnetwork with the highest *t*-test score, and also followed up with two additional suboptimal subnetworks, if their discriminative power was within 2% of the optimal extended subnetwork that has the largest *t*-test score. We iteratively repeated the extension process until the discriminative power of the subnetworks could not be improved by at least 5% through such extension. The entire process of identifying the defense-related subnetwork modules was reiterated for all the seed genes and for all four co-expression networks. Finally, we selected four potential genetic subnetwork modules that are likely to be associated with maize defense response based on their discriminative power for differentiating between the two conditions (*i.e*., inoculation by wild type vs. mutant), as well as by investigating the presence of significant GO terms associated with maize defense system, either directly or partially. In Figures 3 and 4, genes relatively highly expressed in the wild type-infected samples are shown in red, whereas genes relatively highly expressed in the mutant-infected samples are shown in blue. Table 3 shows basic properties of the four identified maize subnetwork modules. As shown in Table 3 the number of genes ranged between 6 and 8 and the number of significant interactions ranged between 5 and 8. Also, the *t*-test statistics scores ranged from 5.1 to 7.2, which were higher than most of the other candidate subnetworks.

**Table 3:** Properties of the potential maize defense subnetwork modules identified by the proposed network-based analysis pipeline

Potential maize subnetworks	number of genes	number of interactions	*t*-test score
Figure 3 module-A	7	6	5.4
Figure 3 module-B	8	8	5.6
Figure 4 module-A	8	8	7.2
Figure 4 module-B	6	5	5.1

### Potential maize subnetwork modules directly associated with maize defense response

Two potential maize subnetwork modules identified by the proposed network-based comparative analysis pipeline contained maize genes whose annotated GO terms were representative terms typically associated with responses to fungi. Figure 3 module-A contained three known maize genes, GRMZM2G001696 T01, GR-MZM2G374971 T01, and GRMZM5G870932 T01, associated with a significant GO term GO:0009817. For this GO:0009817, whose GO term is "defense response to fungus (incompatible interaction)", is defined as "a response of an organism to a fungus that prevents the occurrence or spread of disease". Benjamini-Hochberg FDR *p*-value of the most significant GO term, GO:0009817, for the Figure 3 module-A was 1.25e-06. Figure 3 module-B included three known maize genes, GRMZM2G001696 T01, GRMZM5G870932 T01, and GRMZM5G878558 T01, associated with a significant GO term, GO:0009620. This GO term is "response to fungus" and the definition is "any process that results in a change in state or activity of a cell or an organism (in terms of movement, secretion, enzyme production, gene expression, etc.) as a result of a stimulus from a fungus"; the *p*-value of Benjamini-Hochberg FDR for this term was 5.69e-07. Note that both GO terms are directly related to the defense mechanism against fungal pathogens. It is also possible to view these two subnetwork modules as one whole module since the two modules shared two significant genes such as GRMZM2G001696 T01 and GRMZM5G870932 T01, however it is important to note that they were obtained from different seed genes and on different co-expression networks. This can be viewed as a demonstration of the reliability of the proposed network-based comparative analysis pipeline and its effectiveness in identifying potential maize subnetwork modules associated with maize defense mechanism against fungi.

### Potential maize subnetwork modules indirectly involved in maize defense response

The other two potential maize subnetwork modules identified by our proposed pipeline were not directly associated with typical GO terms involved in defense response against fungal pathogens, but they also exhibited potential relevance to the maize defensive mechanism. Figure 4 module-A contained four known maize genes, GRMZM2G003930 T06, GRMZM2G056920 T03, GRMZM2G095025 T01, and GRMZM5G878558 T01, associated with GO term GO:0046914. This term GO:0046914 is for "transition metal ion binding" and had a *p*-value (for Benjamini-Hochberg FDR) of 4.56e-02. The GO term is described as "interacting selectively and non-covalently with a transition metal ions that is an element whose atom has an incompleted-subshell of extranuclear electrons, or which gives rise to a cation or cations with an incompleted-subshell". For module-B in Figure 4, two known maize genes, GRMZM2G001696 T01 and GRMZM2G085019 T01, were associated with GO:0046686. This GO term is for "response to cadmium ion" and is defined as "any process that results in a change in state or activity of a cell or an organism (in terms of movement, secretion, enzyme production, gene expression, etc.) as a result of a cadmium (Cd) ion stimulus". The *p*-value of Benjamini-Hochberg FDR for this GO term, GO:0046686, was 3.00e-02. For the two GO terms (GO:0046914 and GO:0046686), it is known that transition metals including cadmium (Cd) have a positive effect on plant defense system against the pathogenicity; hyperaccumulation of transition metals tends to reduce the growth of pathogens [26]. Since both GO terms, *i.e*. GO:0046914 and GO:0046686, were all significantly related to transition metals, we can expect the two predicted maize subnetwork modules in Figure 4 to be potentially involved in maize defense system. For the four identified subnetwork modules in Figures 3 and 4, note that GO terms of the other genes other than the above-mentioned genes were either insignificant or not specified.

Finally, we investigated orthologous genes of those genes included in the two identified maize subnetwork modules in Figure 4. We looked for orthologous genes in *Sorghum bicolor *as well as *Arabidopsis thaliana *provided by RGAP (Rice Genome Annotation Project) website (http://rice.plantbiology.msu.edu). During this cross-check, we found orthologous genes of Figure 4 module-A member genes (i) involved in transition metal ion binding, such as AT2G01275, AT2G20030, AT4G28890, AT1G37130, and AT1G77760 for *Arabidopsis thaliana *and as SB03G007810, SB09G030900, SB04G024300, SB04G034470, and SB07G022750 for *Sorghum bi-color *and (ii) also associated with GO:0009610, "response to symbiotic fungus", such as SB04G024300, SB04G034470, and SB07G022750. Moreover, orthologs of the genes in Figure 4 module-B, such as AT1G59500, AT2G23170, and AT4G37390 for *Arabidopsis thaliana *as well as SB01G032020, SB02G038170, and SB03G035500 for *Sorghum bicolor*, were annotated with GO:0010279 "indole-3-acetic (IAA) acid amido synthetase activity". The "IAA amido synthetase" is known to be an important controller for plant defense system [27]. This analysis based on the orthologous genes of the identified maize subnetwork modules in Figure 4 demonstrated that these two maize subnetwork modules may play potentially important roles in maize to defend itself from fungal pathogens.

## Conclusion

In this paper, we proposed a network-based comparative RNA-seq data analysis pipeline specifically focusing on host-pathogen (*F. verticillioides *vs. maize) interactions. *F. verticillioides *is not only detrimental to the host plant maize but also to animals and humans, due to toxic secondary metabolites produced on infested commodities. To investigate their interactions, RNA-seq data from maize inbred B73 inoculated with two different *F. verticillioides *strains (wild type vs. *fsr1 *mutant) was prepared. In order to gain insight into the underlying biological functions and network interactions in maize defense response, we first filtered maize genes, using a cointegration-correlation-expression approach, to identify candidate genes whose activities corresponded to those of selected *F. verticillioides *virulence genes. Subsequently, we predicted the co-expression networks containing these maize candidate genes and searched for potential subnetwork modules likely to be associated with the maize defense mechanism. Based on our pipeline, we identified four potential maize subnetwork modules associated with the defense response against the *F. verticillioides *virulence. The member genes of the identified subnetwork modules showed relevance to defense-associated GO terms, well coordinated expression patterns with each other, and differential expression under the two different conditions (i.e., inoculation with wild type vs. *fsr1 *mutant). As shown in Figure 3, two of the identified maize subnetwork modules were directly associated with maize defense response against the *F. verticillioides *pathogenicity. The other two identified modules, shown in Figure 4, are also likely to be involved in the maize defense system, as they were predicted to be linked to accumulation of transition metals and defense response, and furthermore, as their member genes have orthologous genes in *Sorghum bicolor *and *Arabidopsis thaliana *that are associated with plant defense. Our results demonstrate that the proposed network-based analysis pipeline can improve our understanding of the biological mechanisms that underlie host-pathogen interactions, and that it has the potential to unveil novel genetic subnetwork modules specifically associated with plant defense response.

## Competing interests

The authors declare that they have no competing interests.

## Authors' contributions

Conceived and developed the approach: MK, BJY. Performed the computational analysis: MK. Analyzed the outcome: MK, BJY, WBS. Obtained the experimental data: HZ, WBS. Wrote the paper: MK, BJY, WBS, CW.

## Supplementary Material

Additional File 1 Table S1: Pearson's correlation coefficients between the candidate maize genes and the four selected *F. verticillioides *pathogenicity genes. This table shows how the maize candidates and the representative pathogenicity genes are correlated. Based on the respective coefficients, corresponding maize genes whose Pearsons correlation coefficients were higher than 0.65 (*p*-values less than 0.0035) to each selected *F. verticillioides *pathogenicity gene were considered as candidates. Table S2: Gene IDs and the most significant GO terms of the predicted subnetwork modules shown in Figure 3. This table helps to see the information such as gene IDs and their significant GO terms for the two subnetwork modules in Figure 3. Table S3: Gene IDs and the most significant GO terms of the predicted subnetwork modules shown in Figure 4. This table helps to see the information such as gene IDs and their significant GO terms for the two subnetwork modules in Figure 4. Click here for file
