# Supplementary material for: Computational identification of genetic subnetwork modules associated with maize defense response to Fusarium verticillioides
Source: BMC Bioinformatics. 2015 Sep 25;16(Suppl 13):S12. doi: 10.1186/1471-2105-16-S13-S12 (PMC4597171; doi:10.1186/1471-2105-16-S13-S12)
Supplement: Additional File 1 — Table S1: Pearson's correlation coefficients between the candidate maize genes and the four selected F. verticillioides pathogenicity genes. This table shows how the maize candidates and the representative pathogenicity genes are correlated. Based on the respective coefficients, corresponding maize genes whose Pearsons correlation coefficients were higher than 0.65 (p-values less than 0.0035) to each selected F. verticillioides pathogenicity gene were considered as candidates. Table S2: Gene IDs and the most significant GO terms of the predicted subnetwork modules shown in Figure 3. This table helps to see the information such as gene IDs and their significant GO terms for the two subnetwork modules in Figure 3. Table S3: Gene IDs and the most significant GO terms of the predicted subnetwork modules shown in Figure 4. This table helps to see the information such as gene IDs and their significant GO terms for the two subnetwork modules in Figure 4. [file 1471-2105-16-S13-S12-S1.pdf]

Supplementary Material

"Computational identification of genetic subnetwork modules associated with maize defense response to Fusarium verticillioides"

Mansuck Kim[1], Huan Zhang[2], Charles Woloshuk[3], Won-Bo Shim[2]and Byung-Jun Yoon*[1,4]

[1] Department of Electrical and Computer Engineering, Texas A&M University, College Station, TX, USA
[2] Department of Plant Pathology & Microbiology, Texas A&M University, College Station, TX, USA
[3] Department of Botany & Plant Pathology, Purdue University, West Lafayette, IN, USA
[4] College of Science and Engineering, Hamad bin Khalifa University (HBKU), Doha, Qatar

Email: Mansuck Kim - mk22893@neo.tamu.edu; Huan Zhang - huanzfly@tamu.edu; Charles Woloshuk - woloshuk@purdue.edu; Won-Bo Shim - wbshim@tamu.edu; Byung-Jun Yoon*- bjyoon@ece.tamu.edu;

*Corresponding author

Table S1: Pearson's correlation coefficients between the candidate maize genes and the four selected *F. verticillioides* pathogenicity genes

Gene ID	*FSR1*	*FST1*	*FvVE1*	*ZFR1*
GRMZM2G084110_T01	0.756930303	0.66702942	0.776972261	0.727111432
GRMZM5G878558_T01	0.603550903	0.708903856	0.390741801	0.515528154
GRMZM2G082899_T01	0.813724749	0.75586743	0.872085477	0.797863371
GRMZM2G552314_T01	0.635650564	0.499574296	0.690976931	0.484945923
GRMZM2G130076_T01	0.603146938	0.592570235	0.735590736	0.712373336
GRMZM2G031968_T01	0.609552098	0.724276088	0.656440686	0.635897737
GRMZM2G030465_T01	0.822006112	0.855904143	0.6604346	0.774240388
GRMZM2G032177_T01	0.658625601	0.537971169	0.641863742	0.573446583
GRMZM2G054023_T01	0.631472357	0.649875847	0.773938136	0.774566626
GRMZM5G869635_T01	0.748379433	0.662177558	0.818386486	0.768281628
GRMZM2G082906_T01	0.722012743	0.570541806	0.679616833	0.589510965
GRMZM2G148494_T01	0.727367962	0.476898912	0.53549507	0.500125966
GRMZM2G181039_T01	0.721343187	0.704467703	0.81942655	0.71226227
GRMZM2G301122_T01	0.695157094	0.597879957	0.560220047	0.684232966
GRMZM2G451716_T02	0.753403727	0.644970658	0.561496771	0.654703008
GRMZM2G044306_T02	0.673381813	0.537033001	0.614642157	0.434272494
GRMZM2G097505_T01	0.742005462	0.683729371	0.868935985	0.825820737
GRMZM2G087291_T02	0.653183624	0.558943029	0.668354015	0.668931382
GRMZM2G475014_T01	0.858869307	0.847991217	0.786447554	0.846878005
GRMZM5G815584_T01	0.675426322	0.782114573	0.662778791	0.658687581
GRMZM2G001696_T01	0.696993145	0.708040447	0.578657687	0.659219394
GRMZM2G122543_T01	0.663699561	0.605717854	0.700694836	0.740398907
GRMZM2G475059_T01	0.657166685	0.618504743	0.682898162	0.675953184
GRMZM2G130904_T01	0.679160441	0.706686945	0.716244844	0.768782538
GRMZM2G008607_T01	0.685195868	0.625091733	0.697301457	0.766499433
GRMZM2G082529_T02	0.664881167	0.525968035	0.558621549	0.565332616
GRMZM2G457309_T01	0.703682574	0.553262553	0.675774296	0.593578997
GRMZM2G110276_T01	0.629093129	0.619058636	0.745531592	0.674889528
GRMZM2G703821_T01	0.5988298	0.500423391	0.727085939	0.69506735
GRMZM2G302373_T01	0.629038889	0.564125682	0.664587351	0.665295923
GRMZM5G878660_T01	0.718095079	0.645325624	0.624218234	0.562900673
GRMZM2G056920_T03	0.552156839	0.641908059	0.691522822	0.721224351
GRMZM2G059381_T03	0.703600263	0.656203817	0.559844423	0.713141246
GRMZM2G143854_T03	0.634407925	0.644169807	0.51703887	0.66787454
GRMZM2G466545_T01	0.526061826	0.661500497	0.439461623	0.653368717
GRMZM2G148355_T01	0.776375797	0.831231061	0.788129403	0.854105943
GRMZM2G143669_T01	0.76322338	0.75773503	0.741706407	0.806307219
GRMZM2G143669_T03	0.698778035	0.688332728	0.746758706	0.797698651
GRMZM2G143669_T04	0.696025329	0.76598941	0.825440926	0.89752938
GRMZM2G358827_T01	0.730634406	0.690196219	0.73178106	0.75742563
GRMZM2G076410_T01	0.517531795	0.472826062	0.618370381	0.66342525
GRMZM2G025459_T01	0.727866735	0.6733106	0.874897375	0.755306136
GRMZM2G025459_T02	0.725232313	0.692447968	0.88620822	0.787565934
GRMZM2G312877_T01	0.524137867	0.508210718	0.658765207	0.509001395
GRMZM2G168681_T01	0.793700165	0.739692178	0.889403645	0.800238932
GRMZM2G168681_T02	0.828705329	0.770967448	0.879571391	0.814689966
GRMZM2G324903_T01	0.660466517	0.684812539	0.837635106	0.843819949

Gene ID	*FSR1*	*FST1*	*FvVE1*	*ZFR1*
GRMZM2G047966_T01	0.600537989	0.557314872	0.648833539	0.702650909
GRMZM2G097141_T01	0.741751395	0.650249944	0.608880233	0.644452961
GRMZM2G003930_T06	0.670630613	0.617398534	0.754723346	0.795332694
GRMZM2G049581_T02	0.686517077	0.642834217	0.562776553	0.709472812
GRMZM2G153615_T01	0.6953764	0.579399856	0.697936841	0.68678629
GRMZM2G416965_T01	0.578364376	0.565766703	0.82892706	0.72901181
GRMZM2G028393_T01	0.724445758	0.615274045	0.798027744	0.667612458
GRMZM2G028393_T02	0.717509478	0.619121531	0.780901912	0.672810669
GRMZM2G378106_T02	0.721171428	0.709518905	0.549556184	0.581957036
GRMZM2G091588_T01	0.841582423	0.789648746	0.671951113	0.731160247
GRMZM2G358051_T06	0.676814273	0.528220168	0.558391211	0.543102552
GRMZM5G803873_T01	0.757417441	0.542597914	0.59074325	0.590608115
GRMZM2G073175_T01	0.656323343	0.539221222	0.640113336	0.664567671
GRMZM2G573083_T01	0.596864817	0.550759147	0.527554671	0.664579832
GRMZM2G045809_T01	0.729777044	0.671181391	0.689471754	0.675603683
GRMZM2G131405_T01	0.753557498	0.717066231	0.844165957	0.80935102
GRMZM2G094328_T01	0.578818667	0.653128933	0.687421781	0.673783327
GRMZM2G103897_T02	0.574945732	0.575803189	0.58884351	0.731802872
GRMZM5G802429_T01	0.738644424	0.77980646	0.550500832	0.684261338
GRMZM2G468439_T01	0.667576201	0.59451732	0.797428181	0.674213071
GRMZM2G012460_T01	0.834919922	0.742718316	0.719319072	0.742711734
GRMZM2G173615_T01	0.735641562	0.680774194	0.757952199	0.700531212
GRMZM2G177878_T02	0.613791731	0.567815961	0.661327649	0.691832613
GRMZM5G870932_T01	0.689121123	0.690273523	0.543668587	0.710949213
GRMZM2G102382_T02	0.716469469	0.569888997	0.477898991	0.557821093
GRMZM2G001799_T01	0.656616502	0.74789073	0.441474628	0.603779514
GRMZM2G326270_T01	0.674965192	0.564530003	0.663972758	0.671513884
GRMZM2G136429_T04	0.650669674	0.658949096	0.433352621	0.633422423
GRMZM2G017145_T02	0.655165104	0.600103414	0.643663465	0.692304655
GRMZM2G374971_T01	0.704431949	0.664010583	0.836630723	0.752139686
GRMZM2G108133_T02	0.677886254	0.618816552	0.617033896	0.670272319
GRMZM2G416625_T01	0.698979203	0.663750102	0.763362423	0.712525045
GRMZM2G700192_T01	0.747321127	0.6677126	0.747803813	0.63358545
GRMZM5G803276_T01	0.641441711	0.601610894	0.837220303	0.775741031
GRMZM2G061702_T02	0.641758795	0.686159521	0.678267887	0.745619604
GRMZM2G001708_T01	0.690480813	0.700132847	0.876006537	0.8174485
GRMZM2G014395_T01	0.656099418	0.594465608	0.691421348	0.711801028
GRMZM5G886315_T01	0.623249282	0.599594525	0.643134708	0.711917488
GRMZM2G055898_T01	0.530676023	0.531144381	0.788753568	0.672730672
GRMZM2G033359_T01	0.806247552	0.743766839	0.74072393	0.691141264
GRMZM2G449343_T01	0.668119554	0.695997884	0.525908469	0.578370463
GRMZM2G078090_T01	0.663175318	0.6088028	0.764097556	0.697779732
GRMZM2G701362_T01	0.683505126	0.666069007	0.741688352	0.632726391
GRMZM2G019090_T01	0.740891309	0.658151991	0.67441408	0.709632513
GRMZM2G019090_T02	0.747520561	0.710603434	0.728156646	0.78203392
GRMZM2G332280_T01	0.664762772	0.603152351	0.690267888	0.758794543
GRMZM2G089970_T01	0.697319419	0.623650297	0.789266334	0.74325166
GRMZM2G024695_T01	0.793926841	0.618413986	0.711217323	0.635654171
GRMZM2G034534_T01	0.737011113	0.743269574	0.73543004	0.78017115
GRMZM2G085019_T01	0.624050488	0.532894165	0.665607467	0.674146722
GRMZM2G085019_T02	0.515027295	0.502393669	0.683647839	0.684721948
GRMZM2G095025_T01	0.666206681	0.610979627	0.672982154	0.587305703
GRMZM2G175610_T01	0.444809948	0.490785869	0.629646263	0.69276903

Gene ID	*FSR1*	*FST1*	*FvVE1*	*ZFR1*
GRMZM5G856297_T01	0.623435397	0.734729038	0.692169398	0.782016971
GRMZM2G137535_T01	0.752317533	0.681328233	0.677965107	0.664874607
GRMZM2G366411_T01	0.760789636	0.639626361	0.638959254	0.567181446
GRMZM5G866583_T01	0.743488528	0.6243497	0.717703873	0.673048432
GRMZM2G150276_T01	0.621646515	0.535317809	0.775867558	0.565849485
GRMZM2G152041_T01	0.700919434	0.636363021	0.653748289	0.701961209
GRMZM2G586921_T01	0.684766824	0.619253509	0.733881355	0.685972828
GRMZM2G011662_T02	0.802398389	0.752490731	0.837393171	0.868178365
GRMZM2G011662_T04	0.676662995	0.693870363	0.837057968	0.849248127
GRMZM2G059285_T01	0.680815626	0.605809435	0.773727347	0.721850276
GRMZM2G500106_T01	0.822105916	0.751704711	0.824490509	0.723634492
GRMZM5G830749_T01	0.681258892	0.589307478	0.765051596	0.718539098
GRMZM2G495850_T01	0.760838098	0.684924941	0.699265279	0.728754006
GRMZM2G102737_T01	0.683246618	0.769041828	0.497617617	0.65748818
GRMZM2G384884_T01	0.670754226	0.618475525	0.466236042	0.536410645

Table S2: Gene IDs and the most significant GO terms of the predicted subnetwork modules shown in Figure 3.

Figure 3	Gene ID	Significant GO	
Module A	GRMZM2G001696_T01	GO:0009817	GO:0009817: defense response to fungus
	GRMZM2G056920_T03		
	GRMZM2G095025_T01		
	GRMZM2G097141_T01		
	GRMZM2G102737_T01		
	GRMZM2G374971_T01	GO:0009817	
	GRMZM5G870932_T01	GO:0009817	
Module B	GRMZM2G001696_T01	GO:0009620	GO:0009620: response to fungus
	GRMZM2G003930_T06		
	GRMZM2G044306_T02		
	GRMZM2G102737_T01		
	GRMZM2G136429_T04		
	GRMZM5G803873_T01		
	GRMZM5G870932_T01	GO:0009620	
	GRMZM5G878558_T01	GO:0009620	

Table S3: Gene IDs and the most significant GO terms of the predicted subnetwork modules shown in Figure 4.

Figure 4	Gene ID	Significant GO	
Module A	GRMZM2G003930_T06	GO:0046914	GO:0046914: transition metal ion binding
	GRMZM2G012460_T01		
	GRMZM2G056920_T03	GO:0046914	
	GRMZM2G061702_T02		
	GRMZM2G082529_T02		
	GRMZM2G095025_T01	GO:0046914	
	GRMZM2G136429_T04		
	GRMZM5G878558_T01	GO:0046914	
Module B	GRMZM2G001696_T01	GO:0046686	GO:0046686: response to cadmium ion
	GRMZM2G011662_T04		
	GRMZM2G085019_T01	GO:0046686	
	GRMZM2G091588_T01		
	GRMZM2G378106_T02		
	GRMZM2G449343_T01		
